# Assessing Rapid Visual Perception in Medical Students Trained in Ultrasound

**DOI:** 10.1007/s40670-025-02327-6

**Published:** 2025-02-27

**Authors:** O. Mescher, C. Musick, B. C. Landis, R. Anderson, A. C. Pappas

**Affiliations:** College of Osteopathic Medicine, Rocky Vista University, Ivins, USA

**Keywords:** Ultrasound, Medical Education, Anatomy

## Abstract

Rapid visual perception of ultrasound (US) images was compared between 1st- and 2nd-year medical students at 2 days or 6 months following ultrasound training, respectively. Briefly, US images were presented for 0.2 s, after which, participants were asked to identify the anatomical structure. Modest accuracy was observed among both cohorts. However, accuracy more than doubled for students that engaged with US outside their formal training. Our study demonstrates: 1) an inexpensive method of assessing rapid visual perception in students of US; and 2) that a combination of formal training and extracurricular engagement leads to greater visual expertise for conventional US.

## Background

Ultrasound (US) offers a portable, minimally invasive means of visualizing internal structures in real-time. In recent decades, physicians across a variety of disciplines have begun utilizing US at bedside in rapidly increasing numbers [8]. Consequently, integration of US training within undergraduate medical curricula is steadily on the rise [10, 11].

In conventional US, a patient’s underlying anatomy is depicted as a gray-scale image reflecting the tissue’s inherent echogenic properties. Accurate interpretation of US imaging relies on the observer’s ability to recognize familiar structures based on their echogenic patterns and features. Visual perception, therefore, is 1) crucial for diagnostic accuracy [3, 14], and 2) is a skill that should be emphasized and assessed in learners attempting to gain mastery over US.

The human visual system is capable of rapid, sub-second recognition of familiar objects and scenes, including medical imaging [2]. Kundel and Nodine [5] demonstrated remarkable accuracy among experienced radiologists in detecting abnormalities in chest radiographs presented for 200 ms. Notably, presentation of an image for just 200 ms eliminates the possibility for visual search and limits the observer to a single fixation [4, 6]. Effectively, this requires the observer to rely on “vision at a glance”, rather than “vision with scrutiny”, when deciphering an image [2].

As with other imaging modalities, the value of US ultimately depends on the user’s ability to interpret the images. Consequently, there is a growing market for artificial intelligence image analysis software that can aid in image interpretation and guide clinical decision-making [9, 13, 15]. Less emphasis is being placed on developing visual expertise or assessing visual perception during a clinician’s training.

To gain more insight into the development of visual expertise during US training, we demonstrate a tool to assess rapid visual perception in medical students that have completed a formal, hands-on US training program. Specifically, we ask if students can accurately identify anatomical structures in US images with just a single fixation. Moreover, we ask whether extracurricular engagement with US affects student performance. Our results indicate a modest level of accuracy among medical students with formal US training (27%−37%). However, accuracy more than doubled (80%−90%) for students that engaged with US beyond their formal training. Our preliminary findings suggest that a combination of formal US training with extracurricular US exposure enables medical students to achieve a near maximal level of visual expertise for conventional US.

## Activity

### Participants

This study was reviewed and approved by the Rocky Vista University (RVU) Institutional Review Board (IRB #2022–119) and was conducted in accordance with the ethical standards established by the 1964 Declaration of Helsinki and its subsequent amendments. Briefly, three cohorts of participants were recruited for this study: 1) 1st-year osteopathic medical students (OMS-Is) recruited 2 days after completing 14 h of formal US training, 2) 2nd-year osteopathic medical students (OMS-IIs) recruited 6 months after completing 14 h of formal US training, and 3) graduate students (GS) with no formal US training. All participants were enrolled at RVU at the time of participation in this study. Participation was voluntary. No incentives were provided for participating in the study. All participants had vision corrected to 20/20 for the duration of the task.

### Task, Study Design, and Survey

**Fig. 1:**
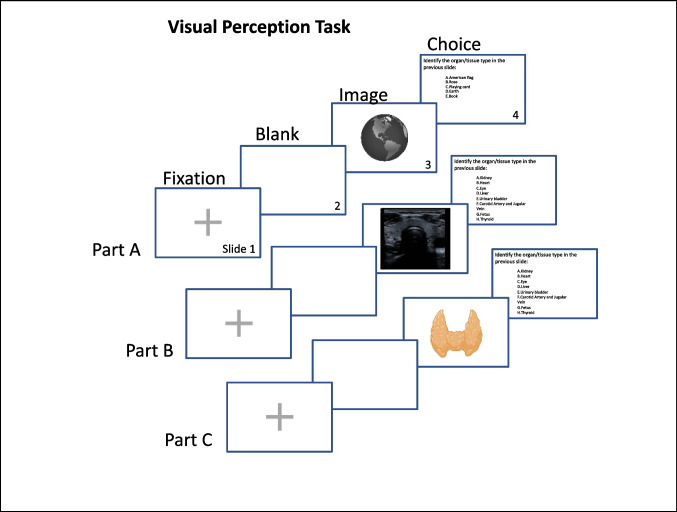
Task and study design. Our custom visual perception task included 25 sequences, each of which contained 4 slides. Part A contained 4 sequences and tested our participants’ ability to identify commonplace objects (ex: globe) depicted as a cartoon. Part B contained 14 sequences and tested our participants’ ability to identify anatomic structures in an US image. Seven anatomic structures (heart, kidney, liver, thyroid, urinary bladder, carotid vessels, and fetus) were tested randomly and twice throughout the task. Part C contained 7 sequences and tested our participants’ ability to identify the same anatomic structures (from Part B) depicted as a cartoon. From the fixation slide, participants left-clicked to initiate the following sequence: blank slide presented for 500 ms, image slide presented for 200 ms, MCQ presented until the participant left-clicked to advance to the next sequence

Our visual perception task was created using Microsoft PowerPoint. Briefly, participants were presented with the following four-slide sequence repeated 25 times throughout the test session: 1) fixation cross, 2) blank screen, 3) image (US or other), and 4) a multiple-choice question (MCQ) asking participants what they had just seen. There were no time constraints on slides 1 and 4. Participants were instructed to left click to either initiate or advance to the next sequence. Slides 2 and 3 advanced automatically after just 500 and 200 ms, respectively (Fig. 1). Participants recorded their responses manually by writing the letter (A-H) corresponding to the structure/object presented during the sequence. During the task, participants were presented with either test or control images. Test images included custom US images of the heart, kidney, liver, thyroid, urinary bladder, and carotid vessels (i.e., common carotid artery and internal jugular vein) obtained using a Mindray MX7 US machine. Our US images were captured by investigators OM, CM, and BL under the supervision of a board-certified radiologist and US instructor, RA. Control images included cartoon images of commonplace objects (ex: book, globe) obtained from Microsoft Office, cartoon anatomical structures (heart, kidney, liver, thyroid, urinary bladder, carotid vessels) created using BioRender, and a 3-D reconstructed US image of a human fetal face obtained from Radiopaedia.org, case courtesy of Servet Kahveci. Each US image was presented at random twice throughout the session. Responses were only marked correct if participants accurately identified the anatomic structure both times. Additionally, only participants that correctly identified the fetal face and control images were included in our analysis. These inclusion criteria minimize the influence of guessing on task performance and ensure that participants can at least recognize one familiar object (i.e., a face) in an US image. Prior to the task, participants completed a brief survey indicating their status (i.e., OMS-I, OMS-II, GS) and whether they engaged with US beyond their formal training (OMS-I and OMS-II only). OMS-I and OMS-II participants were not asked to indicate the amount of time they engaged with US outside of the curriculum.

### Formal US Training

At the time of this study, formal US training during the first-year medical curriculum at RVU consisted of 7 h of hands-on training, 7 h of lecture (14 h total), and 7 exams. Following completion of this training, all osteopathic medical students (OMS-I and OMS-II) would have been exposed to each of the structures tested in our visual perception task. GS received no formal US training.

## Results & Discussion

**Fig. 2:**
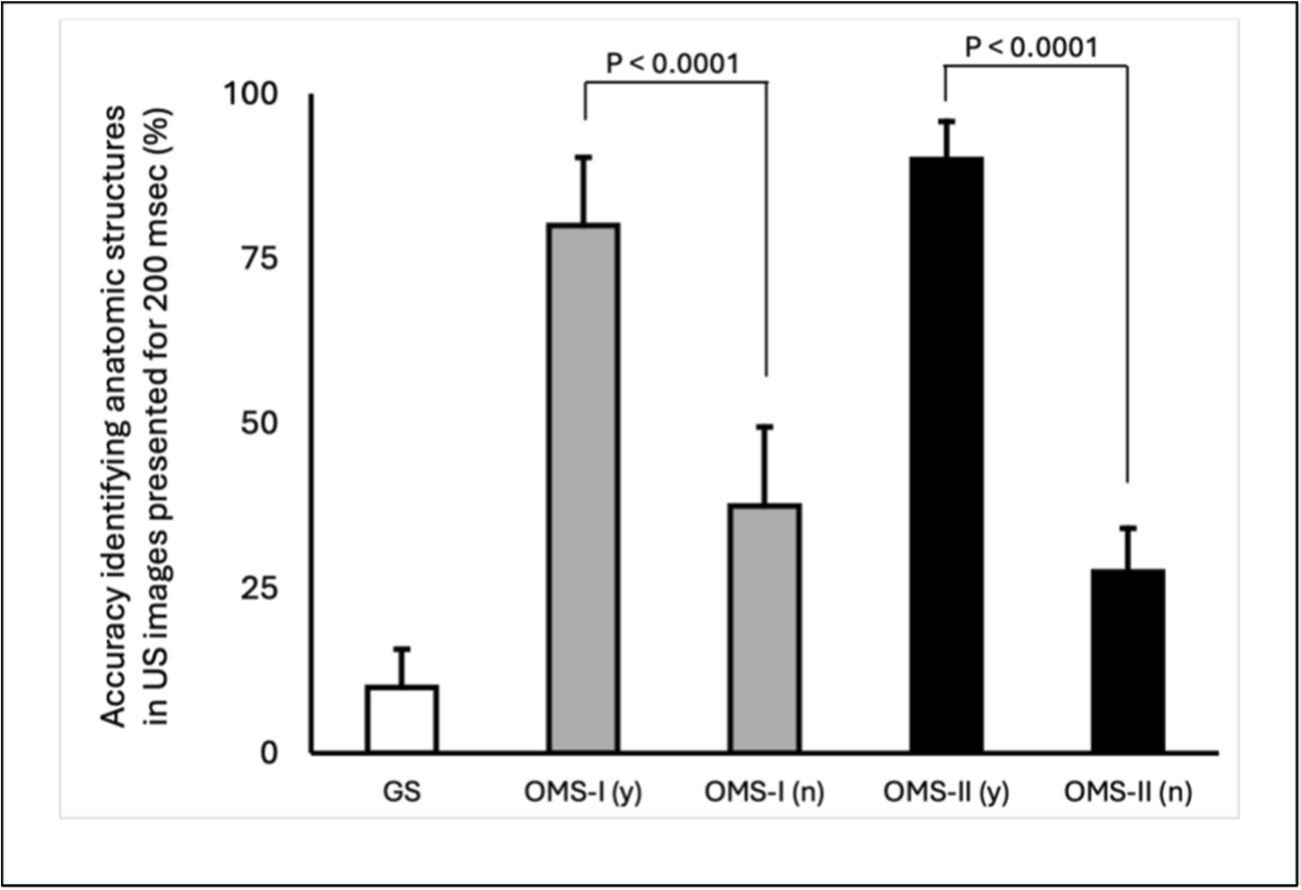
Extracurricular engagement with US significantly enhances accuracy in identifying anatomic structures in US images presented for 200 ms. Medical students that engaged with US outside their formal training (OMS-I [y] and OMS-II [y]) exhibited significantly greater accuracy compared to medical students that did not engage with US outside their formal training (OMS-I [n] and OMS-II [n]), or graduate students (GS) that received no formal training

OMS-I and OMS-II participants were administered our visual perception task 2 days and 6 months after completing their US training, respectively. Initially, we observed similar levels of accuracy identifying anatomic structures in US images between groups (OMS-I: μ = 55.7%, SE = 9.5%, *n* = 14; OMS-II: μ = 48.3%, SE = 9.9%, *n* = 13). However, when groups were split based on engagement with US outside their formal training, we noted a marked difference in student performance. Accuracy was significantly higher among OMS-Is that indicated engagement with US beyond their formal training, compared to OMS-Is that did not (Fig. 2; OMS-I [y] μ = 80%, SE = 10.3%, *n* = 6; vs OMS-I [n] μ = 37.5%, SE = 10.3%, *n* = 8; *p* < 0.0001). This trend was also observed among OMS-IIs (Fig. 2; OMS-II [y] μ = 90%, SE = 5.8%, *n* = 4; vs OMS-II [n] μ = 27.5%, SE = 6.6%, *n* = 9; *p* < 0.0001). OMS-I and OMS-II participants without extracurricular US experience performed significantly higher than the GS group (OMS-I [n] vs. GS *p* < 0.0001; OMS-II [n] vs. GS *p* < 0.00001). GS, who received no formal US training, exhibited the poorest accuracy (Fig. 2; μ = 8.0%, SE = 4.9%; *n* = 5).

All participants exhibited 100% accuracy identifying the 3-D reconstructed US of the fetal face, as well as cartoon images of objects and anatomical structures. These data validate our visual perception task, proving that identification of anatomic structures in US images requires prior training and experience.

We apply an inexpensive method of assessing rapid visual perception to medical students that have completed a formal US training program. We show that 1) formal US instruction during the first year of medical school confers a modest level of accuracy in identifying anatomic structures in US images with just a single visual fixation (up to 40% accuracy), and 2) the ability to identify structures in US images with just a single fixation is significantly enhanced if students engage with US beyond their formal training (80%−90% accuracy).

Conscious recognition of visual stimuli reflects activity across several cortical sites [1, 7, 12], with “gross” visual perception beginning at higher cortical levels, then proceeding in a top-down approach to fill-in the fine details [2]. By eliminating visual search (i.e., vision with scrutiny), our participants cannot rely on top-down processing to identify structures in the images. Rather, they must rely solely on the conceptual “gist” of each image (i.e., vision at a glance). The robust effect of extracurricular engagement with US on task performance suggests that the additional US exposure caused long-term enhancement of neural substrates within higher areas of cortex underlying the visual perception. OMS-Is and OMS-IIs that engaged with US outside their formal training exhibited near maximal level of visual expertise at both time-points tested (2 days, and 6 months post-training, respectively). Remarkably, OMS-IIs that maintained engagement with US beyond their formal training exhibited the greatest accuracy (~ 90%) despite a 6-month lapse in formal training.

We acknowledge the limitations of this study. Sample sizes were relatively small across all groups. Lack of funding prevented more effective recruitment efforts. Moreover, additional information regarding students’ extracurricular engagement with US is presently lacking. Students at our institution can access US machines after-hours and on weekends. Engagement with US under these conditions is unsupervised and therefore cannot be precisely determined. It is also recognized that the OMS-II cohort had more time to potentially engage with US outside of the curricula compared with the OMS-I cohort. Future studies with more detailed survey questions are necessary to determine the nature and quantity of extracurricular US experiences impacting task performance.

Despite the limitations of our study, we demonstrate a visual perception assessment tool that accurately reflects our learners’ level of training and experience in conventional US. Moreover, our task utilizes Microsoft PowerPoint, a staple at most academic institutions. Outside of the cost of acquiring and maintaining sufficient US machines to support an US curriculum, there are few barriers that would prevent an institution from implementing such a tool to assess rapid visual perception in their students.
